# Reducing Humidity Response of Gas Sensors for Medical Applications: Use of Spark Discharge Synthesis of Metal Oxide Nanoparticles

**DOI:** 10.3390/s18082600

**Published:** 2018-08-08

**Authors:** Alexey A. Vasiliev, Andrey E. Varfolomeev, Ivan A. Volkov, Nikolay P. Simonenko, Pavel V. Arsenov, Ivan S. Vlasov, Victor V. Ivanov, Alexander V. Pislyakov, Alexander S. Lagutin, Igor E. Jahatspanian, Thomas Maeder

**Affiliations:** 1 Moscow Institute of Physics and Technology, Dolgoprudny, 141701 Moscow Region, Russia; varfol_ae@mail.ru (A.E.V.); volkov256@yandex.ru (I.A.V.); n_simonenko@mail.ru (N.P.S.); arsenov-pasha@mail.ru (P.V.A.); isvlasov5@yandex.ru (I.S.V.); ivanov.vv@mipt.ru (V.V.I.); thms.maeder@gmail.com (T.M.); 2 NRC “Kurchatov Institute”, 123182 Moscow, Russia; palexv_07@mail.ru (A.V.P.); lagutin_as@nrcki.ru (A.S.L.); 3 ITMO University, 191002 St. Petersburg, Russia; drjie@mail.ru; 4 Kurnakov Institute of General and Inorganic Chemistry, Russian Academy of Sciences, 119991 Moscow, Russia; 5 École Polytechnique Fédérale de Lausanne, CH-1015 Lausanne, Switzerland

**Keywords:** breath test, biomarkers, hydrogen gas sensor, spark discharge synthesis of nanoparticles, minimization of humidity response

## Abstract

The application of gas sensors in breath analysis is an important trend in the early diagnostics of different diseases including lung cancer, ulcers, and enteric infection. However, traditional methods of synthesis of metal oxide gas-sensing materials for semiconductor sensors based on wet sol-gel processes give relatively high sensitivity of the gas sensor to changing humidity. The sol-gel process leading to the formation of superficial hydroxyl groups on oxide particles is responsible for the strong response of the sensing material to this factor. In our work, we investigated the possibility to synthesize metal oxide materials with reduced sensitivity to water vapors. Dry synthesis of SnO_2_ nanoparticles was implemented in gas phase by spark discharge, enabling the reduction of the hydroxyl concentration on the surface and allowing the production of tin dioxide powder with specific surface area of about 40 m^2^/g after annealing at 610 °C. The drop in sensor resistance does not exceed 20% when air humidity increases from 40 to 100%, whereas the response to 100 ppm of hydrogen is a factor of 8 with very short response time of about 1 s. The sensor response was tested in mixtures of air with hydrogen, which is the marker of enteric infections and the marker of early stage fire, and in a mixture of air with lactate (marker of stomach cancer) and ammonia gas (marker of *Helicobacter pylori*, responsible for stomach ulcers).

## 1. Introduction

One of most important obstacles limiting the application of metal oxide (MOX) semiconductor gas sensors for contaminant trace detection is the humidity dependence of the sensor response. This restriction is well pronounced in the case of the application of gas sensors for the analysis of human breath, where the relative humidity of air is not constant and can reach ~100%. Another example is the detection of low concentrations of air pollutants, e.g., hydrocarbons and hydrogen, which are the markers of a starting fire in the smoldering stage [1]. In addition, due to recent sanitation rules, it is obligatory to detect in industrial safety systems 100 ppm of methane. However, according to the datasheet of Figaro Inc. (Osaka, Japan) [2], the response of the sensor to this concentration is ~40%; at the same time, the response to humidity change from 35 to 100% is ~80%. Therefore, the minimum methane concentration which could be measured without special humidity compensation is equal to about 500 ppm within 2σ accuracy. This makes the application of MOX sensors in modern safety systems very difficult.

On the other hand, there is no alternative to the application of semiconductor gas sensors for the detection of low hydrocarbon and hydrogen concentrations. Indeed, the sensors which could be used for the measurement of low hydrocarbon concentrations are nondispersive infrared devices (NDIR), and thermocatalytic and photoionization devices. Optic NDIR sensors are rather selective, but their disadvantage lies in their relatively high detection limit and rather high cost (~100 Euro). According reference [3], the detection error with an NDIR sensor is equal to 1000 ppm. A similar situation can be found in the case of the application of thermocatalytic (thermochemical) gas sensors [4]. The estimates made using these data show that the detection limit of methane is about 1000 ppm.

The detection limit of hydrocarbons for photoionization sensors could be as low as 1 ppb [5]; however, the limiting factors in this case are the relatively high price (~1000 Euro) and short lifetime of the UV lamp. 

Therefore, the minimization of the humidity response of MOX sensors combining low gas detection limit and low price is important for the expansion of gas sensor applications.

According to a recent point of view [6], the humidity response is due to the presence of OH-groups on the surface of the metal oxide. Several methods were suggested to decrease the concentration of superficial hydroxyl groups, for example, high temperature annealing of sensing material, hydrothermal treatment, functionalization by foreign ions, etc.

The first method (that is, high-temperature annealing of the sensing material) is used by different researchers and companies producing gas sensors. The annealing of tin dioxide sensing material at a temperature of 900–1000 °C decreases humidity response, but also decreases the overall sensitivity of the gas sensor due to a dramatic decrease in the specific surface of the material and crystalline growth at temperatures exceeding 700 °C. For example, annealing of SnO_2_ at 900 °C has been shown to lead to a decrease in specific surface from 32.1 to 10.8 m^2^/g [7]. This corresponds to our own experience, which has shown that the annealing of tin dioxide powder at 800 °C for only 15 min decreases specific surface from ~60 to ~10 m^2^/g.

There are two opposite methods to decrease the rate of crystallite growth. The first consists of the application of clean methods of SnO_2_ synthesis leading to material free of inorganic ions—Cl^−^, Na^+^, and others existing in inorganic precursors like SnCl_2_. In this method, tin acetate is used as precursor for the synthesis of tin dioxide powder [8]. Vice versa, 1 wt% Nb doping of SnO_2_ [9] also decreases the rate of crystalline growth of SnO_2_ by a factor of 3–4 at 900 °C. This decrease is due to the segregation of doping ions, and its concentration on the surface of crystallites.

This decrease in the humidity response of gas sensors related with high-temperature calcinations of the sensing material is not the only possible way. Obviously, an opposite approach that is the saturation of the surface with hydroxyl groups can be used as well; such a saturated surface cannot adsorb more water, and this leads to reduced humidity response of the sensor [10]. The authors treated the sensing layer with a 0.04 mol/L solution of H_2_SO_4_, followed by treatment with a 0.1 mol/L of thiocarbamide (2 min). After this, the material was annealed at 600 °C for 1 min. As a result, the influence of humidity in a range from near zero to 95% RH was decreased considerably, by a factor of two.

To decrease the concentration of OH-groups, we used dry synthesis of SnO_2_ material—the basic material for hydrogen and hydrocarbon gas sensors. The idea of this approach consists of the application of spark discharge between metallic tin electrodes in air. As a result, tin evaporates from the surface of electrodes with the formation of airborne particles being oxidized by the oxygen contained in the carrier gas (air). Therefore, it is possible to directly produce airborne particles treated at high temperatures, but without crystallite growth, because airborne particles do not have contact between each other during the treatment process.

We concentrated our efforts on the measurement of the response of the gas sensor to the main gas markers of important diseases. These are low concentrations of hydrogen, marker of enteric infections leading to the formation of hydrocarbons and hydrogen [11] with concentrations in a range from 20 to 100 ppm; lactate, which is marker of stomach cancer [12]; and ammonia gas, a marker of the presence of *Helicobacter pylori*, responsible for stomach ulcers [13].

## 2. Materials and Methods

**Figure 1:**
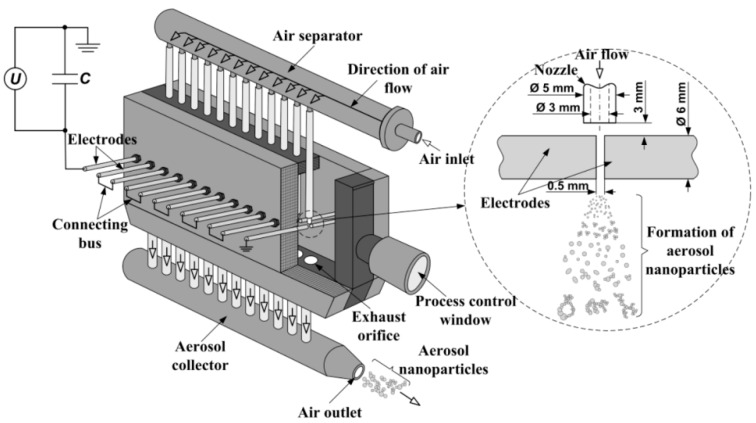
Scheme of the multi-spark discharge generator.

The gas sensing material was synthesized by spark discharge. This method is very promising for producing various nanoparticles from any parent bulk materials (electrodes) with satisfactory conductivity (ρ < 0.2 Ω∙cm) [14]; this condition is fulfilled for all metals and some semiconductors such as doped Si, Ge, and Sb [14,15,16,17]. In this work, we used a custom-built multi-spark discharge generator [18,19] containing 12 pairs of serially connected cylindrical electrodes powered by a 12 nF capacitor charged by a high-voltage source (Figure 1). 

The electrodes made of pure tin (purity ~99.95%) with a nominal diameter of 6 mm were aligned axially at a distance of 0.5 mm and blown continuously with dried (20–30%) clean air at a rate of 15 m/s. The optimum values of the source output voltage and the pulse repetition frequency providing a reasonable combination of the mean size (<100 nm) and the production rate (>1 g/h) of airborne nanoparticles were found to be 4.5 kV and 2.5 kHz, respectively. Substantial increase in these values of frequency and voltage leads to the formation of arc discharge instead of sparks, whereas their decrease results in the drop of the production rate of nanoparticles. Details of the optimization procedure are described elsewhere [20,21].

The airborne nanoparticles were deposited onto an air filter made of porous stainless steel. In order to reduce the metallic phase content, the as-synthesized powder was annealed in air atmosphere as follows: (i) heating up to 610 °C at a constant rate of ~5 °C/min; (ii) keeping at 610 °C for 2 h.

The analysis of EDX spectra of the annealed powder showed that the concentration of possible impurities (such as Fe, Ni, Cu, and Cr, being the constituents of the components of the spark discharge generator chamber) is below the detection limit of the EDX method, which is about 0.1%. According to the results of this analysis, the powder is composed of only tin and oxygen atoms.

The sensing ink was made by mortar mixing of pure SnO_2_ powder (without any catalyst decoration) with a solution of ethyl cellulose in terpineol. This vehicle is usable as a rule for the preparation of inks for screen printing [22]. The viscosity of the ink was adjusted for easy deposition of the ink by dispensing.

The design of the microheater chip used for the deposition of the sensing material was described in detail earlier, for example, in [23]. Briefly, the sensor is an alumina substrate with dimensions of 2.5 × 0.5 × 0.1 mm suspended in TO8 packaging by using 5 mm long, 20 μm thick Pt wires. The platinum-based composite microheater (sheet resistance about 2 Ω/square) is formed on one side of this substrate, whereas Pt pads and the sensing layer are formed on the other side. The distance between pads is about 0.3 mm, and the thickness of sensing layer is about 20 μm. The droplet of the sensing ink was deposited on the sensor chip by dispensing with the use of a needle. The deposited ink was dried at 300 °C (15 min) and then fired at 720 °C (15 min).

The phase composition of the materials used for the fabrication of the sensing layer was retrieved from X-ray diffraction (XRD) spectra measured with a D8 DISCOVER (Bruker, Karlsruhe, Germany). The particle size distribution was determined from transmission electron microscopy (TEM) images obtained with a JEM-2100 (JEOL, Tokyo, Japan). The specific surface area was estimated by the BET method with the use of a TriStar 3000 (Micromeritics, Norcross, GA, USA). The size distribution of airborne nanoparticles was measured in the output flow just prior to their deposition with a DAS 2702 aerosol spectrometer (AeroNanoTech, Moscow, Russia). The gas sensitivity was studied by using a commercial Microgas-F instrument (Intera, Moscow, Russia) [24]. This instrument consists of four independent gas channels equipped with pressure stabilizers and mass flow controllers. These lines are (1) a line of premixed gas mixture in cylinder (in our case—H_2_/air mixture); (2) a line of pure air used for diluting the premixed gas mixture; (3) a line of pure air passing after the mass flow controller through a bubbler filled with distillated water, with the humidity of air in this line close to 100% RH; and (4) a line passing through a diffusion source of gas, which was not used in these experiments. Appropriate and simultaneous adjustment of gas flows in lines (1), (2), and (3) enables the independent setting of desirable concentrations and humidity in the output gas line, where the gas mixture is obtained by mixing gas from lines (1), (2), and (3) without any risk of affecting gas concentration by solution/dissolution of gas in water. The response time of the mass flow controllers was about 1–2 s.

Some preliminary tests with only humidity and cross-sensitivity tests with lactose and ammonia gas were performed using a simple bubbler filled with water and a solution of lactose or ammonia gas in water. In this last case, the gas concentration was controlled by setting the concentration in the water solution. Such an approach permits the simulation of conditions existing in human body. The gas was exchanged in this case by a hand-operated valve.

## 3. Results and Discussion

**Figure 2:**
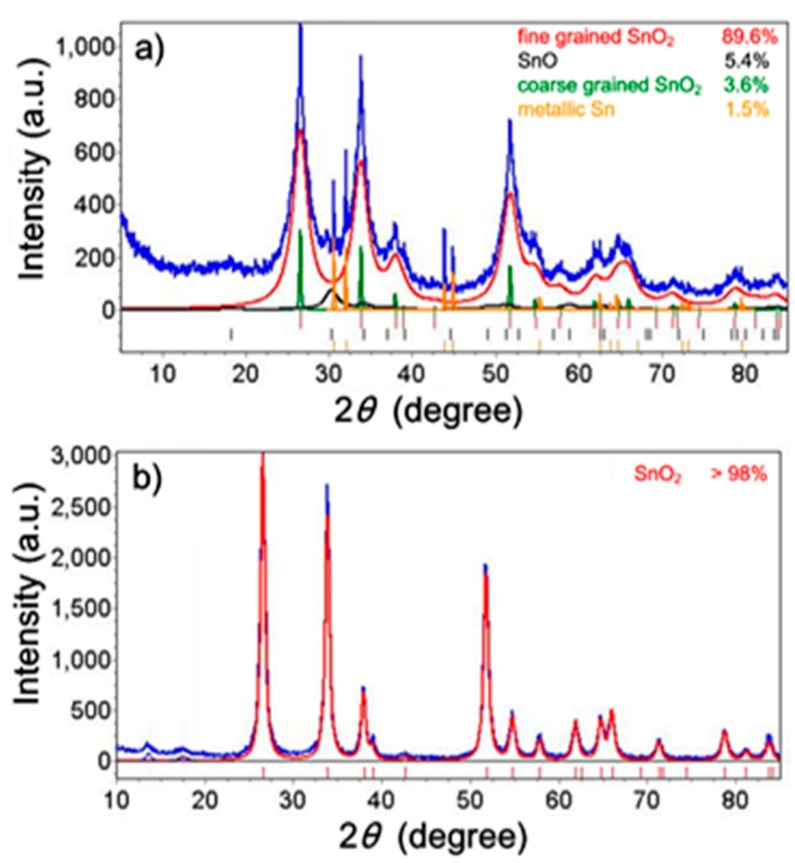
Results of XRD phase analysis of as-synthesized (**a**) and annealed (**b**) powders. The measured spectra are shown in blue. Other spectra calculated by the Rietveld method correspond to crystalline phases indicated in the upper right area of each figure.

From XRD phase analysis, it was found that the as-synthesized powder comprised the following crystalline phases: SnO_2_ (93.2 ± 0.5 wt%), SnO (5.4 ± 0.1 wt%), and metallic Sn (1.5 ± 0.1 wt%). The annealed (610 °C) powder contains only the SnO_2_ phase (>98%) and traces of unidentified phases. The measured XRD spectra together with the calculated spectra of the constituent crystalline phases are presented in Figure 2a,b.

**Figure 3:**
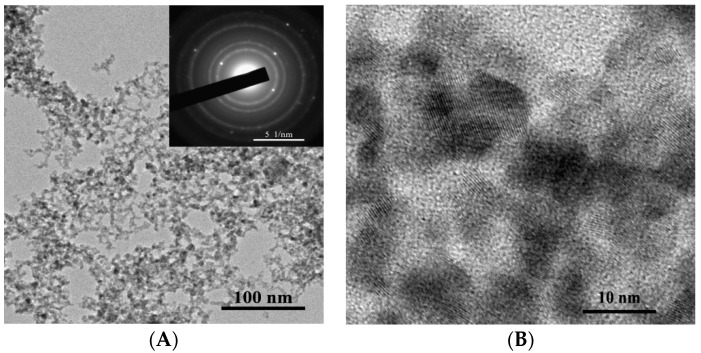
TEM images of the as-synthesized nanoparticles at different magnifications (**A**,**B**); electron diffraction pattern (inset of figure (**A**)).

**Figure 4:**
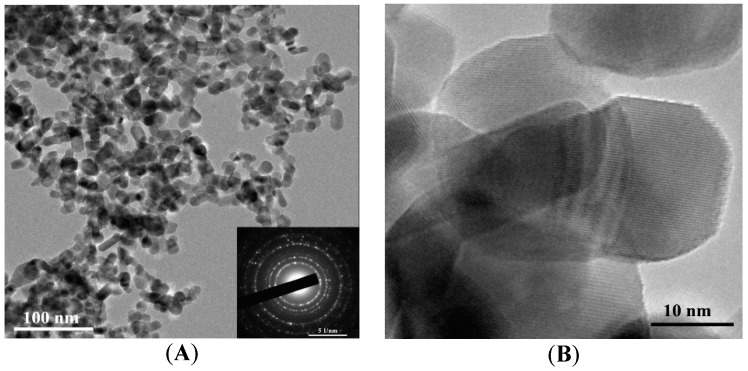
TEM images of the nanoparticles annealed at 610 °C at different magnifications (**A**,**B**); electron diffraction pattern (inset of figure (**A**)).

The as-synthesized powder is represented by primary near-spherical particles and their aggregates of irregular shape (Figure 3A,B). The annealed powder used for the formation of the gas-sensing layer is represented by near-spherical and slightly elongated particles (Figure 4A,B) possessing certain surface faceting.

**Figure 5:**
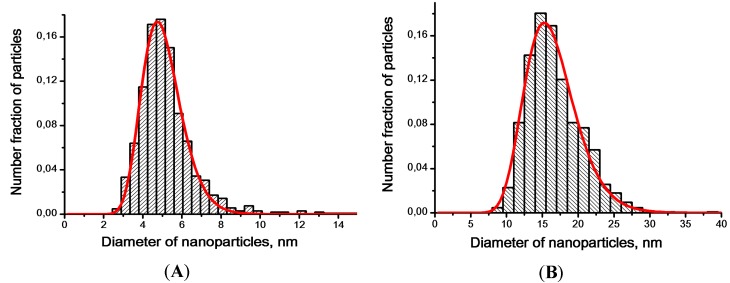
Particle size distribution of as-synthesized SnO_2_ powder (**A**) and of the powder after annealing at 610 °C (**B**).

The histogram obtained from the analysis of TEM images of primary particles constituting the as-synthesized powder is well described by a log-normal distribution with the modal size of about 4.7 nm (Figure 5A). The histogram corresponding to the particles constituting the annealed powder is well described by a log-normal distribution with the modal size of about 15.3 nm (Figure 5B). According to the electron diffraction patterns (insets of Figure 3A and Figure 4A), particles in both materials have crystalline structure; this is also evidenced by TEM images at high magnification (Figure 3B and Figure 4B).

The specific surface area of as-synthesized and annealed powders was found to be about 130 and 40 m^2^/g, respectively. The reduced specific surface area of the latter is due to the increased mean particle size caused by recrystallization which occurred during the annealing. The results of the characterization of as-synthesized and annealed powders are summarized in Table A1, Appendix A.

**Figure 6:**
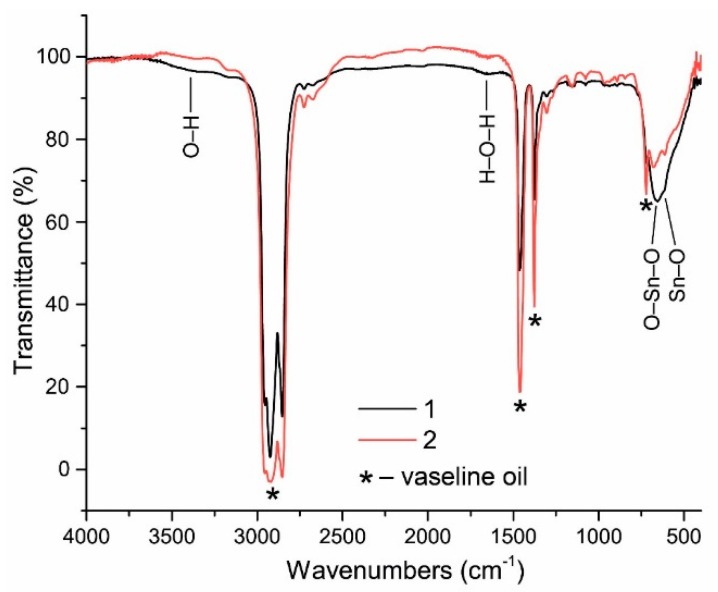
FTIR spectra of as-synthesized (1) and annealed (2) powders measured at room temperature.

FTIR spectra of as-synthesized (1) and annealed (2) powders are presented in Figure 6 (vaseline oil was used as a binding substance in order to form a thin uniform layer of the material for measuring transmittance of infrared radiation). As can be seen from this figure, the content of OH-groups (valent vibrations in the range 3400–3500 cm^−1^) and adsorbed water (deformation vibrations in the range 1600–1650 cm^−1^) in the as-synthesized powder is very low. After the annealing, the content of OH-groups becomes negligible. For comparison, the content of OH-groups in chemically synthesized tin dioxide materials is much higher as confirmed by IR spectroscopy [25,26].

**Figure 7:**
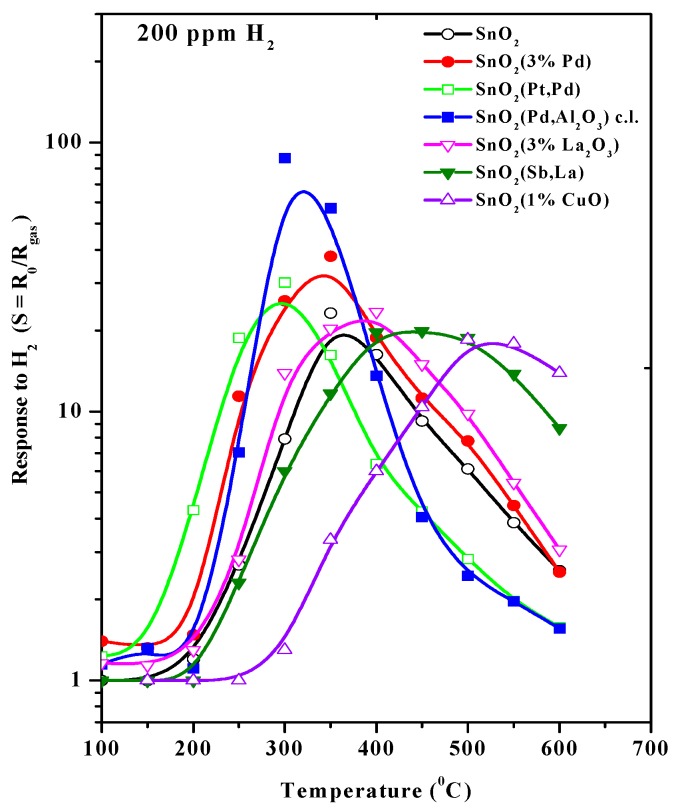
Response of the sensor based on SnO_2_ with different decorations to 200 ppm of H_2_ as a function of sensor temperature. Reprinted with permission from author [27].

The response to hydrogen of gas sensors with a tin dioxide sensing layer deposited onto a microheater used in this work is presented in Figure 7 [27]. The author of this work, V.V. Malyshev, investigated in detail sensing layers with different decorations over a wide range of humidity. In particular, the optimal temperature for pure SnO_2_ obtained by the sol-gel method is close to 350 °C.

We used these results to set the working temperature of our hydrogen sensor at about 450 °C, according to the recommendations [27], to decrease the humidity response, because the maximum response to humidity also lies at about 350 °C.

**Figure 8:**
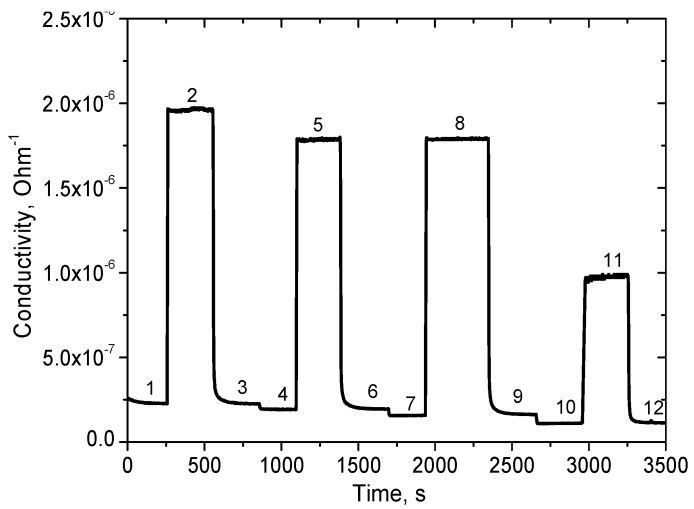
Sensor response to 100 ppm of hydrogen at different values of humidity: 1, air, RH80; 2, 100 ppm H_2_, RH80; 3, air, RH80; 4, air, RH60; 5, 100 ppm H_2_, RH60; 6, air, RH60; 7, air, RH40; 8, 100 ppm H_2_, RH40; 9, air, RH40; 10, air, RH30; 11, 100 ppm H_2_, RH30; 12, air, RH30.

The resistance of the sensor under study was measured at different concentrations of hydrogen in the air and different values of humidity of the air. The typical response of the sensor to 100 ppm of hydrogen is presented in Figure 8.

**Figure 9:**
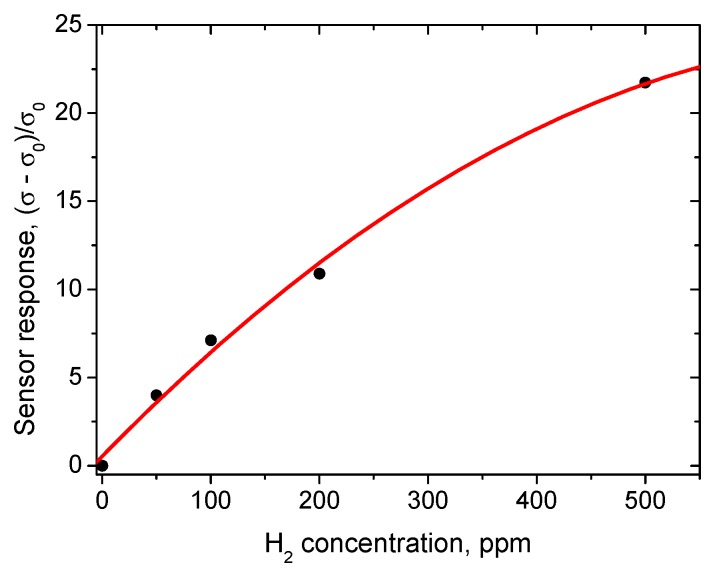
Sensor response to different concentrations of hydrogen at RH60.

**Figure 10:**
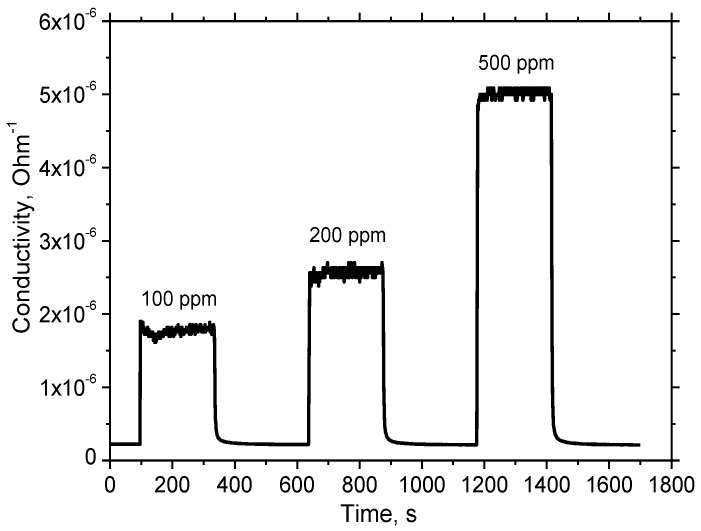
Sensor response as a function of hydrogen concentration in a range up to 500 ppm at relative humidity of 60%. σ_0_ is the sensing layer conductivity at zero hydrogen concenetration, σ is the conductivity in gas mixture H_2_/air.

At the working temperature of 450 °C, the resistance drop does not exceed 20% in the humidity range of 40–100% (Figure 8). The sensor responses to different concentrations of hydrogen in the range from 100 to 500 ppm are given in Figure 9. The corresponding plot showing sensor response as a function of hydrogen concentration demonstrates the usual power law (Figure 10), which is typical of such sensors [28].

**Figure 11:**
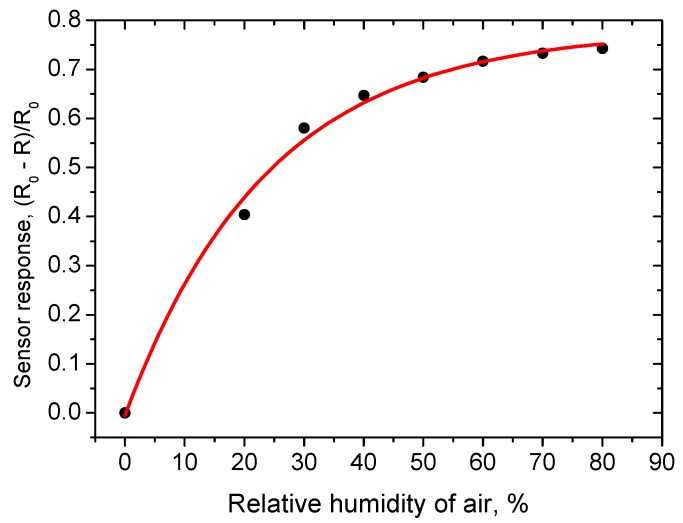
Relative change of resistance as a function of relative humidity. R_0_ is the sensing layer resistance at zero humidity, R is the resistance in humid air.

At the same time, the sensor response (Figure 8) to a hydrogen concentration of 100 ppm exceeds a factor of 8 in the humidity range from 40 to 80% RH. This hydrogen response is close to the response of a sensor based on the material synthesized by the sol-gel method (Figure 7). It is necessary to note that under real sensor exploitation conditions, the detection limit of the sensor is determined mainly by the variation in ambient humidity of air, if special attention is not paid to compensate these variations. Therefore, taking into account that the drop of the resistance caused by humidity variation does not exceed 20% in a range from 40 to 100% RH, the H_2_ detection limit is about 1 ppm (3σ). The drop of the sensing layer resistance as a function of humidity of ambient air is presented in Figure 11.

Therefore, excellent, very fast responses of the sensor to hydrogen (~1 s) and water vapors (~10 s) were observed. This especially concerns the response time to changing humidity, because this property enables easy compensation of the humidity dependence of the gas sensor response by means of electronics.

Whereas a response time to changing hydrogen concentrations equal to several seconds and even less was previously observed by researchers (for example, [29,30]), the typical response time to changing humidity is much longer. It reaches usually several minutes or even several tens of minutes and more.

**Figure 12:**
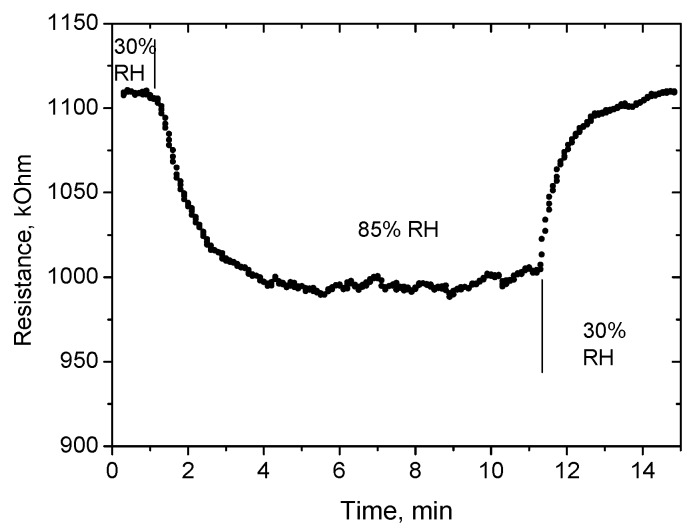
Results of preliminary tests of response and recovery time after change in humidity 30–85–30% RH. Sensor temperature is 450 °C.

**Figure 13:**
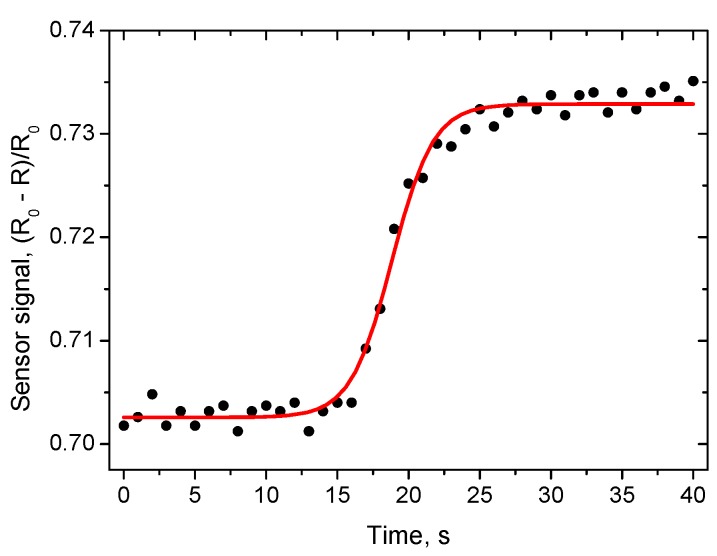
Response time of the sensor to change of humidity: air, RH70 ⇒ air, RH80; Sensor signal is calculated as (R_0_ − R)/R_0_, where R_0_ is the sensing layer resistance in dry air and R is resistance in air with current humidity. Response time to changing humidity is ~10 s (τ_90_—time necessary to reach 90% of final value of conductivity).

Preliminary tests of response and recovery time to changing humidity were performed using simple bubblers filled with distillated water (output humidity in small gas chamber with volume of ~10 cm^3^ is 85% RH) and with a saturated solution of MgCl_2_ (humidity of 30% RH). These results are presented in Figure 12. More detailed results of the measurement of the response time to humidity change from 70 to 80% RH are presented in Figure 13. The response time τ_90_ (time necessary to reach 90% of the final value of resistance) was found to be about 10 s. The recovery time is longer, equal to ~1 min (Figure 12).

**Figure 14:**
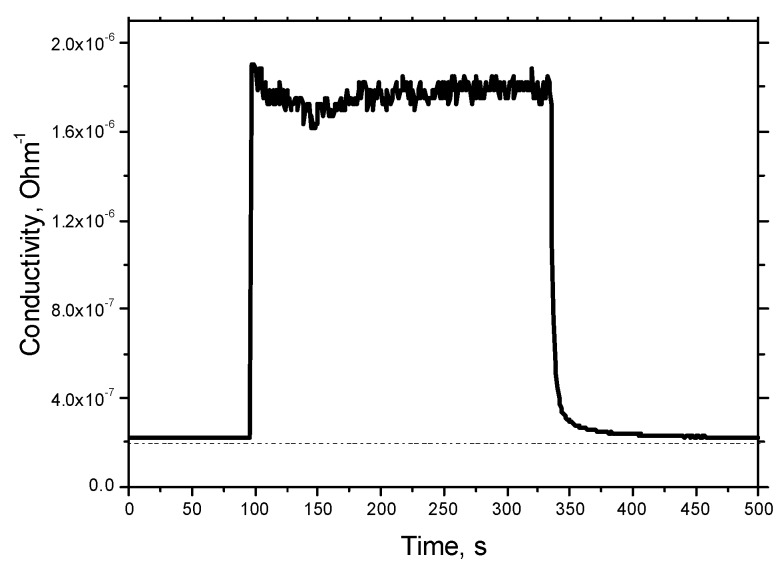
Response/recovery time to 100 ppm of hydrogen in air at working temperature of 450 °C and at relative humidity of 60% (time interval between points—2 s).

Response and recovery of the sensor to 100 ppm of H_2_ is presented in Figure 14. The response time was found to be about 1–2 s, with recovery about 10 s. In this work, no special efforts were undertaken to find true response and recovery times similar to those described in [29]. We used mass flow controllers with intrinsic time of about 1–2 s, and other standard equipment. Therefore, these values of characteristic time are, in fact, a superposition of the true response time of the sensor, the response time of the mass flow controller, and the time necessary to substitute gas in the gas sensor, and are a superior limit of the response/recovery time. 

More interesting, from our point of view, is the analysis of reasons which could lead in our experiments to a very short response/recovery time to changing air humidity.

In another paper [31], the authors analyzed the response time of a tin-dioxide-based sensor to changing humidity. They have shown that the humidity response consists of two parts: fast and slow ones. The fast part of the humidity response (~20% of amplitude) lasts a few seconds, whereas the slow part of the response (~80% of amplitude) takes >5 min.

This result is typical of tin dioxide gas sensors; we also observed similar behavior in tin dioxide sensing material prepared by the sol-gel method. The most important difference obtained for the sensing material fabricated by the spark discharge method developed in this work is the absence of the slow part of the humidity response.

In our opinion, this difference is related with the properties of tin dioxide. Indeed, tin dioxide is not only a semiconductor material. It is, in fact, a mixed conductor having electron and superficial proton conductivity [32]. This proton conductivity seems to be responsible for the slow part of the humidity response (Figure 13). In our case, we do not observe the slow part of the humidity response due to the low density of hydroxyl groups in the material obtained in the spark discharge. 

The mechanism of mixed electron–ion conductivity of tin dioxide will be the goal of our further detailed research; however, the same peculiarities of tin dioxide behavior, well known to all researchers working with tin dioxide sensor material, confirm this conclusion. Among these peculiarities is the shape of the response curve to reducing gases at increasing and decreasing gas concentration. In the first moment after concentration (for example, H_2_) increase, the conductivity of the sensing material is considerably higher than in equilibrium state. Another fact confirming this suggestion is the nonsymmetric behavior of the sensing layer after a change of testing voltage polarity. This behavior is similar to that observed during the electrode polarization. These processes are to be studied in detail in our further research.

The response to hydrogen of the sensor based on tin dioxide material obtained by the spark discharge method was compared with the response to some physiologically important compounds: lactate and ammonia gas. These gases together with hydrogen are markers of gastroenterological diseases, as it was mentioned in the introduction.

To perform these measurements, we used bubbling of carrier air through glass filled with an appropriate solution. This was done to simulate conditions existing in human body. The concentrations of the solutions were equal to 1 mol/L (lactate) and 0.1 mol/L (ammonia). The results of the measurements are presented in Table 1.

Obviously, the hydrogen response of the sensor based on pure tin dioxide obtained by spark discharge is much higher than that to other physiologically important gases. Such sensors can be used as components of electronic noses for noninvasive diagnostics of gastroenterological diseases.

The investigation of long-term stability of the sensor demonstrated that the reproducibility of the response is within 5% during a 120-day test.

## 4. Conclusions

Advanced metal oxide materials with reduced sensitivity to water vapors were investigated. To decrease the concentration of OH-groups, we used dry synthesis of SnO_2_ material. The spark erosion of tin electrodes in air atmosphere results in the formation of airborne tin oxide nanoparticles. It was demonstrated that it is possible to free the surface from hydroxyl groups, to reconstruct the surface of tin dioxide particles in a way that prevents chemisorption of new hydroxyl groups by the surface, but to do this without strong crystallite growth and a strong decrease in target gas sensitivity. This is feasible because in the spark process, tin dioxide particles are separated from each other by air gaps in the process of formation. The sensor shows the outstanding characteristics of response. Very fast and stable response of the sensing material to humidity (~10 s) and hydrogen (~1 s) enables the correction of the results of gas concentration measurements using an additional humidity sensor. Thus, the metal oxide materials synthesized by spark discharge have outstanding properties in comparison with conventional metal oxide sensors prepared by wet sol-gel processes. Such a material is very promising for the development of gas sensors for the fast detection of hydrogen and for monitoring of hydrogen in electronic nose systems to be used for the diagnosis of gastroenterological diseases and, as well, for safety monitoring systems applied in industry. In the latter case, it can be used as a humidity-independent sensing element, for example, for early detection of fire.

## Figures and Tables

**Table 1:** Cross-sensitivity of the sensor based on spark-discharge-synthesized tin dioxide to some physiologically important gases at temperatures taken for the measurements of hydrogen response of the sensor (450 °C).

	Pure Air RH 30% (R30)	Pure Air RH 85%	Air + Lactate RH 85% *	Air + NH_3_ RH 85% **
R, kΩ	1090	990	760	1010
Response *** (Rg − R_30_)/R_30_, %	-	8 ± 1	30 ± 5	7 ± 1

* Lactate concentration obtained by bubbling air through 1 mol/L solution of lactate in distillated water. ** Ammonia concentration obtained by bubbling air through 0.1 mol/L solution of NH_3_ in distillated water. *** Sensor response to different gases is measured as (Rg − R_30_)/R_30_, where R_30_ is sensing layer resistance at 30% relative humidity, and Rg is the resistance in the appropriate gas mixture.

## Appendix A

**Table A1:** Results of characterization of as-synthesized and annealed powders.

Type of Powder	Phase Composition (XRD)	Mean Crystallite Size (XRD) nm	Specific Surface Area (BET), m^2^/g	Modal Particle Size (TEM), nm	Effective Particle Size *, nm
As-synthesized	Fine-grained SnO_2_ tetragonal (89.6 wt%)	4	130	4.7	7
	SnO tetragonal (5.4 wt%)	4			
	Coarse-grained SnO_2_ tetragonal (3.5 wt%)	70			
	Sn tetragonal (1.5 wt%)	>200			
Annealed	SnO_2_ tetragonal (>98 wt%)	15	40	15.3	21
	Unidentified phases (<2 wt%)	–			

* Calculated via specific surface area.
